# New Perspectives for Resistance to PARP Inhibitors in Triple-Negative Breast Cancer

**DOI:** 10.3389/fonc.2020.578095

**Published:** 2020-11-25

**Authors:** Ye Han, Xiaopeng Yu, Shuqiang Li, Ye Tian, Caigang Liu

**Affiliations:** ^1^ Department of Oncology, Shengjing Hospital of China Medical University, Shenyang, China; ^2^ Department of Biomedical Informatics, College of Medicine and Biological Information Engineering, Northeastern University, Shenyang, China

**Keywords:** PARPi, BRCA, resistance, TNBC, DNA damage repair

## Abstract

Poly (ADP-ribose) polymerase (PARP) inhibitors are a therapeutic milestone exerting a synthetic lethal effect in the treatment of cancer involving *BRCA1/2* mutation. Theoretically, PARP inhibitors (PARPi) eliminate tumor cells by disrupting DNA damage repair through either PARylation or the homologous recombination (HR) pathway. However, resistance to PARPi greatly hinders therapeutic effectiveness in triple-negative breast cancer (TNBC). Owing to the high heterogeneity and few genetic targets in TNBC, there has been limited therapeutic progress in the past decades. In view of this, there is a need to circumvent resistance to PARPi and develop potential treatment strategies for TNBC. We present, herein, a review of the scientific progress and explore the mechanisms underlying PARPi resistance in TNBC. The complicated mechanisms of PARPi resistance, including drug exporter formation, loss of poly (ADP-ribose) glycohydrolase (PARG), HR reactivation, and restoration of replication fork stability, are discussed in detail in this review. Additionally, we also discuss new combination therapies with PARPi that can improve the clinical response in TNBC. The new perspectives for PARPi bring novel challenges and opportunities to overcome PARPi resistance in breast cancer.

## Introduction

Poly (ADP-ribose) polymerase (PARP) has been a therapeutic target for the treatment of breast cancer genes 1 and 2 (BRCA1/2) protein-deficient tumor cells since 2005 (1). The interacting mechanism of BRCA and PARP is a genetic deficiency resulting in a synthetic lethal effect due to the functional loss of two genes leading to cell death, while a mutation or defect in either of the two single genes facilitates cell viability. Based on this synthetic lethality theory, PARP inhibitors (PARPis) were developed that increased the sensitivity of the synthetic lethal effect in cancer cells harboring *BRCA1/2* mutations. PARPis have ultimately improved the treatment for patients with mutant BRCA breast and ovarian cancers.

Breast cancer (BC) is the most lethal disease in women due to its high incidence and mortality worldwide. Triple-negative breast cancer (TNBC), which lacks estrogen receptor (ER), progesterone receptor (PR), and human epidermal growth factor receptor 2 (HER2) expression, is one of the most aggressive types of BC and is characterized by rapid recurrence, early metastasis, and poor prognosis. Due to the high heterogeneity and few genetic targets in TNBC, therapeutic progress in the past few decades has been limited. It is reported that about 20–30% of TNBC patients have a proven *BRCA1/2* mutation. Moreover, the molecular signatures associated with “BRCAness” greatly widen the population of *BRCA* mutations or defects (2). Accordingly, BRCA carriers with TNBC are presumably sensitized to DNA damage treatment; however, the clinical outcome is not as expected (3); only 20–40% of the patients benefit from PARPis and are alive five years after diagnosis. The majority of the TNBC patients still experience early relapse and distant metastasis due to ineffective treatment (4). Therefore, for effective treatment and control of TNBC, we urgently need further discussion regarding circumvention of resistance to PARPi and the development of promising treatment strategies. In this review, we aim to present the scientific progress and explore the underlying mechanisms of PARPi resistance in TNBC.

## DNA Damage Repair Pathways

**Figure 1:**
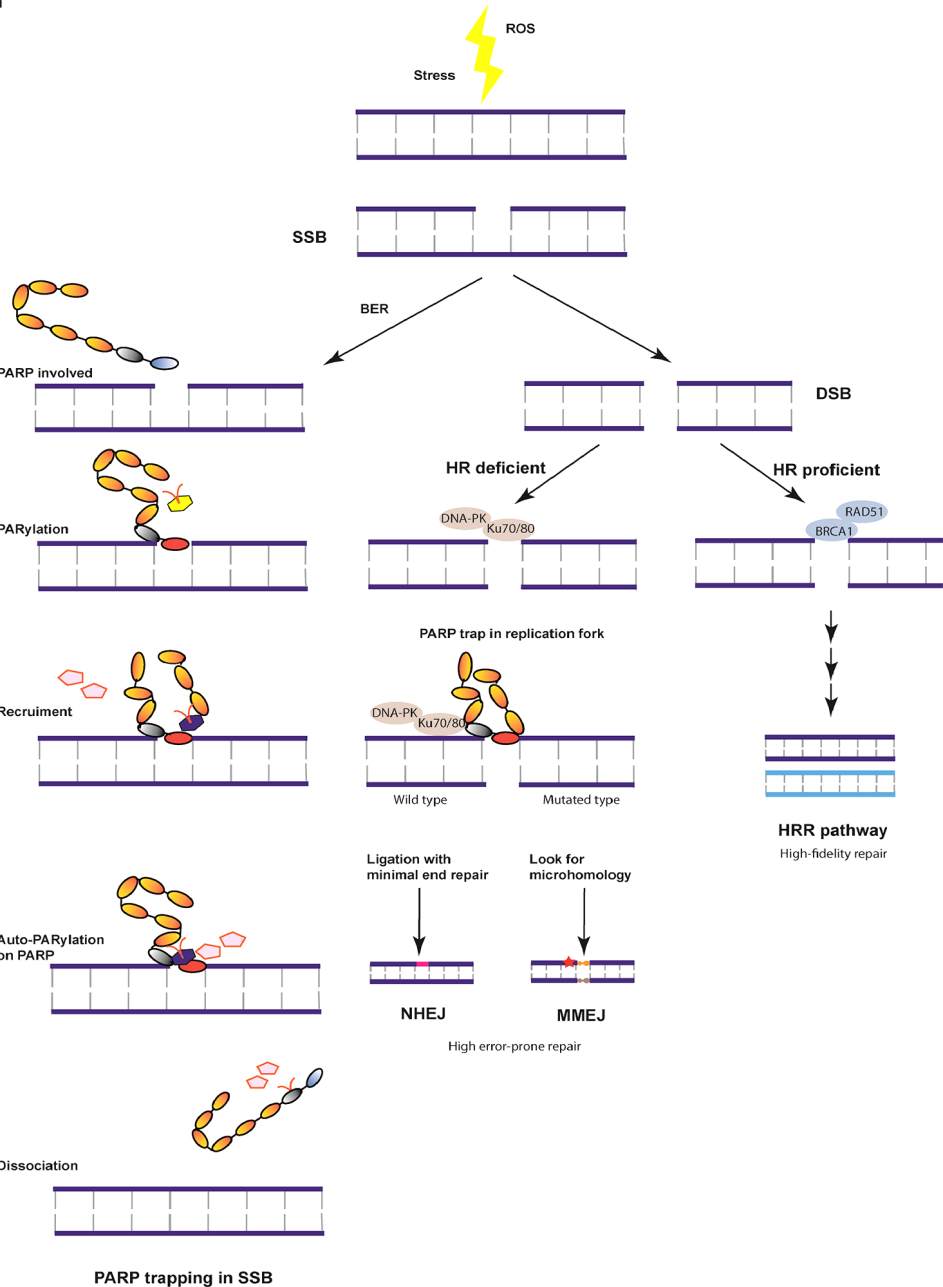
Overview of the DNA damage repair pathway. DNA damage is induced in the S/G2 phase due to stress or reactive oxygen species (ROS). The most stable DNA single-stranded break (SSB) is repaired with the help of base excision repair (BER) and BRCA by poly (ADP-ribose) polymerase (PARP) trapping and dissociation. Otherwise, DNA double strand break (DSB) is formed, which can be repaired by three independent pathways under different mechanisms. With homologous recombination (HR) proficient genes, DNA undergoes high fidelity repair at cell cycle checkpoints. In case of HR deficiency, the damaged chromatin ligation is mediated by minimal end repair or microhomology, both of which are unstable repair pathways resulting in cell death or tumor development.

DNA damage is detected and repaired by DNA single-strand break (SSB) and DNA double-strand break (DSB) repair pathways (5). Compared with the more stable SSB repair pathway, the DSB repair pathway includes two prominent pathways: the homologous recombination (HR) and non-homologous end-joining (NHEJ) pathways. In addition to the HR and NHEJ pathways, the homology-independent repair pathway, named alternative end-joining pathway (Alt-NHEJ) or microhomology-mediated end-joining (MMEJ), can mediate DNA repair at the broken chromatin terminal using distant microhomologies. HR is the classical repair pathway with high fidelity in the mid S and G2 phases of the cell cycle and involves key proteins such as BRCA1 (6). BRCA1 is an active protein in SSB and cell cycle regulation, while BRCA2 assembles the essential recombination enzyme RAD51 and controls the repair process. Among women, about 5% have been detected to have BRCA1/2 deficiency (7). *BRCA1* mutation is more closely associated with TNBC, whereas *BRCA2* mutation is predominantly linked to ER/PR-positive tumors (4). The MRN complex (MRE11, RAD50, and Exo1) is steadily recruited and phosphorylated by BRCA and 53BP1 in the HRR pathway of DNA damage repair. Alternatively, several dynamic enzymes such as Ku70/80, DNA-PK, and XRCC4 are recruited to initiate PARP trapping at the replication fork through the NHEJ pathway (8). To maintain genomic stability, the HRR pathway is the primary choice for DNA damage repair. Of note, the complementary pathways of NHEJ or MMEJ lead to genomic and chromosomal instability, which tends to cause somatic mutations or tumor cell death (**Figure 1**). HR deficiency, leading to genomic instability or accumulated mutations, is the core mechanism for treating TNBC by PARPi.

## BRCA1/2 Mutation in TNBC

BRCA1 was detected by comparing between familial early-onset breast cancer with genomic chromosome 17q21 more than 30 years ago (9, 10). *BRCA1/2* functions as the driving gene in HR repair of DNA double-strand breaks (DSBs). Mutations in the *BRCA1* gene are associated with a high frequency of associated cancer, and the mean accumulative risk for driving breast cancer was estimated to be more than 50% for *BRCA1* carriers by the age of 70 years (11). The *BRCA1* mutation is often accompanied by a *TP53* mutation, based on the examination of clinical samples (12), and increases the risk of breast cancer development (13). Importantly, BRCA1 deficiency can be induced by epigenetic silencing through promoter hypermethylation, which is a leading cause of BRCA1-defective cancer (14). Of note, several genes involved in the HRR pathway, including *BRCA2* and *RAD51*, play a role in tumor development and are implicated in HR deficiency when mutated or defective (15). The genomic instability in the process of DNA repair caused by defective HR has been proven to behave in a similar manner as a *BRCA1/2* mutation, which mediates NMEJ or cell death (16). Due to the importance of PARP1 for the bulk of PARylation upon DNA damage, RNAi-mediated depletion or single RNA-mediated mutation of PARP1 induces synthetical lethality in BRCA1/2-deficient cancer cells. The chromosomal instability of HR-deficient tumors sensitizes the cells to DNA damaging drugs and is one of the mechanisms for BRCA1/2-deficient tumors. Patients harboring *BRCA1/2* mutations exhibit a higher risk of developing ovarian, prostate, and other cancers. PARPi treatment exhibits cytotoxic effects in tumors with *BRCA1/2* mutations or BRCAness, and is a promising targeted therapy based on two main mechanisms: synthetic lethality and PARP trapping (17).

## Demethylation Agents of BRCA1/2

Inactivation of BRCA1 or BRCA2 is associated with a pattern of genome-wide mutations known as signature 3, which is strongly associated with breast cancers. Carriers with BRCA1 mutations develop large numbers of rearrangement signature 3 small tandem duplications (18). Studies on breast cancer have shown that epigenetic silencing of RAD51C and BRCA1 by promoter methylation is strongly associated with signature 3 and is highly enriched in basal-like breast cancers (18). Demethylation drugs are synthetic molecules that modulate the activity of epigenetic proteins, such as DNA methyltransferases, interferon microRNAs, and histone methyltransferases. The DNA modification is methylated by DNMTs by adding a methyl group to the 5-position of cytosine of CpG dinucleotides. DNMT inhibitors, including nucleoside analogs (5-zaz-2′-deoxycytidine or decitabine), antisense oligonucleotides (ASO98), low molecular weight molecules (RG108, Procainamide, Disulfiram), are in clinical trials to understand the underlying mechanisms in demethylation of DNMTs. Hydralazine (CAS No.304-20-1; Sigma-Aldrich) is a primary DNMT inhibitor that binds CpG island sequences and interferes with translocation of DNMTs along the sequences. The combination of hydralazine and thiazolidinedione has apoptotic and antiproliferative effects in TNBC cells (19). Histone methyltransferase (HMT) inhibitors reduce hypermethylation of oncosuppressor genes like BRCA1 and prevent development of breast cancer (20, 21). S-adenosylmethionine analogs are the first inhibitors targeting HMT and other enzyme classes, but they have low specificity that limits their use (22). DZNEP, an inhibitor of KMTs and EZH2, significantly decreases H3K27me3 on the *SRC3* gene in MDA-MB-231 cell lines (23). Panobinostat (LBH589), an inhibitor of overexpressed HDACs in cancer, is specifically effective in gene regulation alone and in combination with other treatments (24). Vorinostat, an HDAC inhibitor, is the first to be approved by the Food and Drug Administration (FDA) and it is at an advanced stage of clinical usage by binding the HDAC catalytic site (25). Resveratrol, an HDAC inhibitor, modulate epigenetic methylation negatively and acetylation positively by restoring expression of *BRCA1, p53* and *p21* genes in breast cancer cell lines (26, 27).

## BRCAness Molecular Trait

*BRCA1/2* mutations are one of the basic genetic features of TNBC associated with a deleterious prognosis and sensitization of tumor cells to PARP inhibitors and platinum-based chemotherapy. Interestingly, a large population of TNBC patients showed a response along with improved survival rates in PARPi clinical trials (28). Researchers have investigated the high-grade genomic instability in non-BRCA-mutated breast cancer. Although these tumors carry no *BRCA* mutation, they show homologous recombination deficiency and are, therefore, called BRCAness tumors. The “BRCAness” is a special phenotype that is not derived from a genetic mutation or deficiency but shares the biological functions of germline *BRCA* mutations (29). The basic mechanism of *BRCA1/2* mutations is the deficiency of BRCA1/2 function in the homologous recombination process during the high-fidelity repair of DNA double-strand breaks (30). The common somatic *BRCA* mutations and copy number variations in TNBC lead to biological deficiency similar to that in germline *BRCA1/2* mutations. In addition, the aberrant expression of BRCA-related proteins disturbs the biological function of BRCA1, which underlies the existence of a complex network involving BRCA genes. Furthermore, homologous repair-related genes such as *TP53*, *MSH6*, and *PTEN*-mutated genes have been demonstrated to be mutated and the driver genes in BRCAness-associated TNBC (31). Theoretically, detection of gene mutations associated with HR deficiency is highly recommended for diagnosing “BRCAness” and predicting the sensitivity to PARPi in TNBC. However, mutational signatures are not as stable as genomic changes but a “scar” on the genome caused by consecutive DNA damage attributable to diverse factors including previous systemic therapy, and this hinders their acuity as a marker for BRCAness (32). Consequently, fresh biopsy for HR status and functional assays for diagnosing BRCAness are necessary. An additional important mechanism is the damage caused to the complex network of DNA damage repair by the natural genomic instability of TNBC, which inversely enhances the sensitivity to PARPi. *RAD51* is a eukaryotic gene that assists in the repair of DNA double-strand breaks. Its mutation has been proven to be a marker of genomic instability and BRCAness (33).

The putative “BRCAness” induced by DNA methylation of the BRCA1 promotor has been repeatedly detected by multiple trials. Epigenetic silencing of the BRCA1 promotor with DNA methylation is theoretically a solid rationale for BRCAness (34). However, in clinical studies, patients with DNA methylation of BRCA1 or with low levels of BRCA1 mRNA did not show a better response rate, progression-free survival (PFS), or overall survival (OS) to carboplatin or PARPis. Conclusively, BRCAness is generally defined as breast tumors with sensitivity to DNA repair deficiency due to various mechanisms other than *BRCA1/2* germline mutations (35).

## Other Gene Mutations in the HRR Pathway

TNBC is characterized by chromosomal instability resulting from homologous recombination repair (HRR) pathway deficiency (HRD) (36). The common causes of HRD include germline BRCA1/2 mutations, BRCA gene promoter methylation, and any genetic mutations of the HRR pathway (37). Some mutated-genes disrupting HRR pathway, including ATM, ATR, PALB2 and CHEK1/2, are the cortical elements that induce irreversible DNA damage and lead to synthetical mortality due to PARPi in TNBC treatment settings (38). A series of genes, including ATM, RAD51, PALB2, MRE11, RAD50, NBN, and the Fanconi anemia proteins, interact with BRCA in DNA damage repair (39). Mutations in PALB2, ATM, RAD50, MRE11, and NBN are involved in hereditary cancers (40). The PALB2 mutation carriers have a 50% risk of breast cancer development over their whole life (41), while ATM mutation carriers have a higher risk of developing breast, pancreatic and prostate cancers (42).

## Mechanism of PARP1 Action

The PARP family comprises 17 nucleoproteins with four domains of interest: a DNA-binding domain, a caspase-cleaved domain, an auto-modification domain, and a catalytic domain (43). When PARP detects DNA double stand breaks, it initiates a polymeric adenosine diphosphate ribose (polyADP-ribose or PAR) chain with NAD^+^ as the substrate (44). The highly conserved PAR polymer reaches as long as 200 nucleotides and transfers one unit to target proteins, whereas PARP1, PARP2, PARP3, and PARP5a characteristically add repeated ADP-ribose units (45). The PARP-initiated post-translation modification in DNA-single strand breaks is called PARylation, which interferes with many cellular activities (46). PARP senses the single-stranded DNA breaks (SSBs) by activating unligated fragments as a trigger for the replication of unperturbed S-phase cells (47). PARP1 is the main responder to DNA damage in the repair pathway, which initiates almost 90% of PAR chains through PARylation catalyzed by PARPs (48). The catalyzed PAR chains initiate the DNA repair process by recruiting a series of targeted DNA repair effectors and chromatin remodeling effectors (49). A special auto-PARylation process restores the catalytical state to an inactive conformation by releasing PARP1 from DNA. Other PARPs such as PARP2 and PARP3 are involved in the base excision repair (BER)/SSB repair pathway and the NHEJ pathway, repectively (50). *PARP1*, as a repair gene, is implicated in multiple DNA repair processes including the pathways of nucleotide excision repair, NHEJ, MMEJ, HR repair, and DNA mismatch repair (51). PARP1 is one of the six essential enzymes responsible for the highly error-prone DNA repair pathway MMEJ (52). High expression of PARP1 usually increases MMEJ and genomic instability, along with highly inaccurate repair leading to mutation or cancer. Therefore, PARP1 is reportedly overexpressed in multiple cancers including those involving *BRCA1/2* mutation, neuroblastoma, ovarian cancer, human papilloma virus-infected oropharyngeal carcinoma, Ewing’s sarcoma, and colon cancer (53, 54). PARP-induced genomic instability is an independent characteristic of tumor development.

In normal cells, PARP1 inhibition induces only the failure of SSB repair but not DSB repair because of efficient BRCA proteins. However, repeated SSBs are inclined to stall and collapse the replication fork, triggering the DSB repair pathway. The mutated *BRCA1/2* DNA lacks the functions of the DSB repair pathway, leading to the NHEJ repair process. NHEJ is an error-prone DNA repair pathway that may result in chromosomal instability, cell cycle arrest, and apoptosis (55). The complicated repair process explains synthetic lethality as one of the main mechanisms of PARP inhibitors in BRCA1/2-deficient or HR chromosomal-instable tumors. The other mechanism of PARPi is the allosteric conformational change that traps PARP1 and PARP2 onto the DNA lesions to exert a different cytotoxicity, which is named as PARP trapping (56). PARP1 trapping is not independent of PARylation, as the core step of PARPi mechanism is prevention of auto-PARylation. PARPis exhibit different types of catalytic inhibition of PARP trapping by direct interactions of the drugs with PARP NAD^+^ at various binding sites, which illustrates the different potencies of PARPis in clinical trials. The details regarding the direct tight binding of PARPi and NAD^+^ are only partially reported and need further molecular and chromosomal studies.

## Distribution of BRCA1/2 and Response to PARPi

BRCA1/2 genes play a critical role in genome integrity and sustaining chromosomal stability for their primary function of DNA repair, cell cycle control, chromatin remodeling and transcriptional regulation. Thus, mutations in BRCA1/2 result in aberrant expression of proteins and genomic instability, which leads to tumorigenesis (57). Carriers of BRCA1 mutations have a 70–80% chance of developing breast cancer, while those carrying BRCA2 mutations have a 40–84% risk of breast cancer (58). In a pan-cancer analysis of BRCA1 and BRCA2 genomic alterations using hybrid captured-based comprehensive genomic profiling, the fraction of BRCA1/2 altered biallelic cases was 68.7%, which is highly associated with elevated genome-wide loss of heterozygosity (gLOH) (59). In clinical trials carriers bearing biallelic BRCA1/2 alterations with elevated gLOH exhibit a therapeutic vulnerability targetable by PARPi. The first BRCA1 mutation, c.2368A > G, causes a change from threonine to alanine at position 790 (p.Thr790Ala) and presents a unique behavior in the clinical development of Afro-descendant women. The second mutation, c.2876G > A, produces a switch in position 959 from arginine to lysine (p.Arg959Lys). The p.Glu1345Lys mutation interferes with the formation of the BRCA1-CBP/P300 complex, resulting in dysfunction of suppressor genes that BRCA1 possesses. Carriers bearing BRCA1, but not BRCA2, mutations present a great amount of rearrangement signature 3 small tandem duplications. Cancers with BRCA1 or BRCA2 mutations exhibit substantial numbers of rearrangement signature 5 deletions (18). The p.Val859Ser*22 mutation of BRCA2, is likely pathogenic and the cause of tumor development, as it generates an early stop codon at position 881 of the BRCA2 protein and truncates proteins with deleterious activity (57). Evidence supports BRCA1/2 mutation as a biomarker for PARPi sensitivity in primary and metastatic breast cancer. Tumors with deficiency of HR are susceptible to PARPi, which is signified by mutations of DNA repair genes including BRCA2, ATM, CHEK2 and PALB2 (60).

As a substantial proportion of patients bearing BRCA1/2 mutations are ER positive, the PARPi response is evaluated according to genetic status. Some studies show similar impairment of the HRR pathway in ER-positive and -negative tumors among carriers with loss of heterozygosity of the wild-type allele BRCA1 (61, 62). Only a small fraction of clinical data validates ER-positive carriers with BRCA1/2 mutations response to PARPi well (63–65).

To broaden the clinical usage of PARPi it is plausible that we evaluate the potential efficacy of PARPi with siRNA/shRNA knockdown libraries or computational methods in conjunction with *in-vitro* responses involving a subpopulation of TNBC patients. A novel approach is used to predict response to PARPi by defining sensitivity and resistance using the DNA damage response and identifying gene predictors with gene set and pathway enrichment analysis (66). The researchers created a unique 63-gene signature, including *RPC1, RPC3, RPC4, RPA1, APEX1, PCNA, PCLB* and *FEN1*, with an overall accuracy of 86%. These genes are associated with PARPi sensitivity, BRCAness, HR or DNA damage response pathways, and there is a need to identify the gene signature using with a larger cohort of PDXs to predict PARPi sensitivity in wild-type TNBC (67).

## PARP Inhibitors Approved in Breast Cancer

PARP inhibitors have a tortuous history in clinical trials. In 1971, the first PARP1 inhibitor was named as nicotinamide by Clark et al., followed by 3-aminobenzamide by Prunell and Whish in 1980 (68, 69). Nicotinamide and benzamide act as competitive substrates to NAD by taking up the catalytic PARP by formation of hydrogen bonds. PARPis are screened by high catalytic activity with a low pharmacodynamic IC50 range, as well as high efficiency of inhibiting PARP1 and PARP2 in clinical trials. Over the past decades, only four PARP inhibitors, namely, olaparib, rucaparib, niraparib, and talazoparib, were originally approved for use as a single agent by the European Medicines Agency (EMA) in the European Union and the FDA in the United States for ovarian cancer (70). Based on the basic scaffolds of PARPis, the new-generation PARPis are selected and classified as follows: phthalazinone and tetrahydropyridophthalazinone act as a scaffold for olaparib and talazoparib; benzimidazole and indazole carboxamide for veliparib and niraparib; and tricyclicindole lactam for rucaparib (71–75). However, only two kinds of PARPis are approved as targeted therapy for breast cancer.

## Olaparib and Talazoparib

Olaparib (AZD-2281, MK-7339, Lynparza) was the first PARP inhibitor that entered clinical trials and was investigated as a targeted therapeutic agent for breast cancer involving BRCA1/2 mutation. On the basis of the results of the randomized phase III study NCT02000622 on patients with metastatic breast cancer with germline BRCA mutation, olaparib tablet (PFS of 7.0 *vs.* 4.2 months; hazard ratio 0.58) was approved by the FDA in 2018 as a second-line treatment for human epidermal growth factor receptor-2 (HER2)-negative metastatic breast cancer (46). Talazoparib (TALZENNA, Pfizer Inc.) exhibits the highest PARP1 trapping efficiency and the most rigid structure (76). On the basis of the strong preclinical findings from the ABRAZO phase II study, talazoparib was analyzed in the phase III EMBRCA study (NCT01945775), showing favorable results (PFS of 8.6 *vs.* 5.6 months; hazard ratio 0.54). It was approved by the FDA as a treatment option for patients with germline BRCA-mutated, advanced HER2-negative breast cancer (77). Niraparib (ZEJULA, Tesaro) and Veliparib (ABT-888) are still under clinical trials for the treatment of TNBC.

## Mechanism of PARPis Resistance

**Figure 2:**
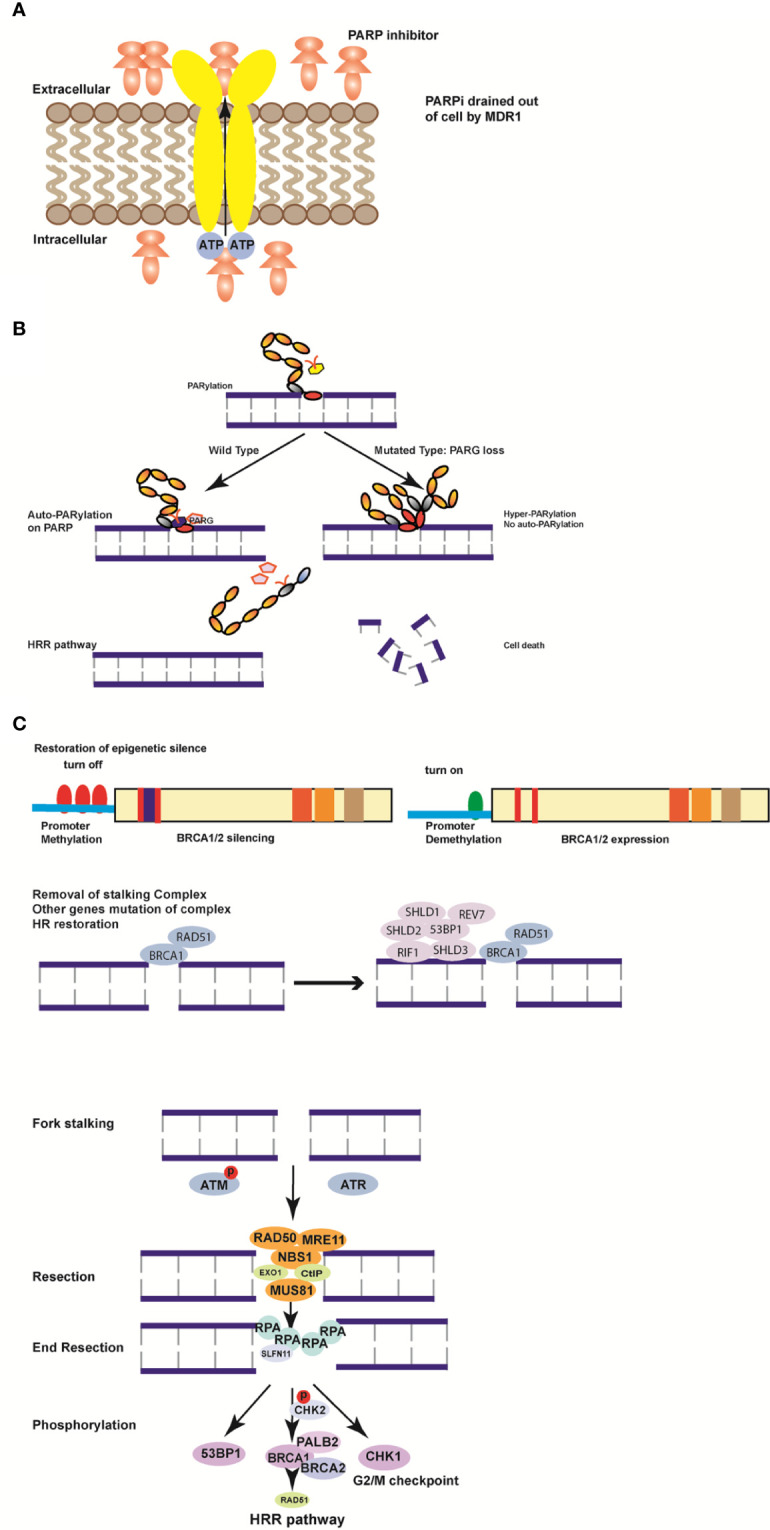
Mechanisms of resistance to poly (ADP-ribose) polymerase inhibitors (PARPi). **(A)** MDR1 is formed to excrete PARPi actively out of the cells to weaken the efficiency of PARPi. **(B)** Loss of PARG induces hyper-PARylation of damaged chromatin and cell death. **(C)** Hypermethylation of *BRCA1/2* genes in the promoter region turns on the function of BRCA1/2 to restore the homologous recombination (HR) pathway. **(D)** Removal of the stalling complex restores the HR pathway and induces PARPi resistance. The restoration of fork stability is a DNA damage response (DDR). ATM or ATR kinase is recruited by the stalled replication fork in response to DNA double strand breaks. By phosphorylating DNA damage sensor complexes such as MRE11, MUS81, NBS, RAD50, Exo1, and CtIP, the replication fork is restored by end resection through RPA prolongation. Subsequently, downstream genes including Chk1, BRCA1, and 53BP are phosphorylated, and chromosomal stability is restored to activate the HR pathway.

Although germinal BRCA 1/2 mutations are markers of PARPi sensitivity, 40–70% of the patients remain unresponsive or resistant to PARPi. The strikingly high percentage of PARPi resistance has led to extensive research to elucidate the resistance mechanisms (78–80). The mechanisms have been categorized into four types to improve the understanding about PARPi use and limitations (**Figure 2**).

## Reduced Cellular Availability of PARPi

Drug-efflux transporter genes (*ABCB1a, ABCB1b*, and *ABCB2*) encoding multidrug resistance protein 1 (MDR1) were found to be highly expressed and exhibited epithelial-to-mesenchymal transition phenotypes in a PARPi resistant cohort in a murine model of BRCA1-mutated breast tumors (81). The *ABCB1* gene is commonly demonstrated to be upregulated after chemotherapy due to chromosomal translocations in ovarian and breast cancers. Elizabeth and colleagues detected frequent transcriptional fusions of *ABCB1* increasing substrate chemotherapy sensitivity in relapsed breast cancer. They proved that MDR1 inhibition in a fusion-positive ovarian cancer cell line increased sensitivity to paclitaxel more than 50-fold (82). Based on the fact that all PARPis are MDR1 substrates, PARPis are increasingly excreted by the transporters outside the tumor cells, thus inducing PARPi resistance. Therefore, the combination of a MDR1 inhibitor and PARPi in germinal BRCA mutated breast cancer remains a promising but uncertain therapeutic option.

## Disruption of PARP1 and PARG Proteins

DNA damage repair is a critically regulated procedure, which is composed of PARylation catalyzed by PARP proteins and auto-PARylation removal by poly (ADP-ribose) glycohydrolase (PARG). PARP1 is always the dominant protein responsible for the entrapment of cellular PARylation upon DNA damage due to its high nuclear aggregation and its initiation of synthetic lethality with HR deficiency (83–85). The PARP trapping degree can be determined by either nuclear-soluble or chromatin-bound fraction distribution of PARP1. PARP entrapment caused interaction between DNA-strand breaks as well as topoisomerase I (TOP1)-processed ribonucleotides and unligated Okazaki-fragments of DNA replication (86, 87). PARP1 initiates DNA damage repair by covalent PARylation and needs repulsion between auto-PARylation and the DNA strand to finish the repair procedure. PARP1 mutations resulting in the loss of trapping of the protein on DNA are the main cause of PARP resistance (88). Genome-wide and high-density CRISPR-Cas9 screening in PARPi resistant tumor cells identified that PARP1 intramolecular interactions might influence PARPi-mediated cytotoxicity. All detected mutations either within or outside the DNA-binding domain altered PARP1 trapping and induced PARP1 resistance in BRCA1/2-mutated cases (89). PARP1 trapping loss independently led to a PARPi resistant cohort due to mutation-induced cytotoxicity of PARP1 and established it as the mechanism of resistance.

The catalytic inhibitory effect is important for DNA repair, which involves relevant PARG and PARP inhibitors. PARG is responsible for the degradation of PAR chains, which is a prerequisite for the DNA damage repair procedure. In a murine BRCA2-mutated cell line, PARG loss was identified as a mechanism for PARPi resistance (90). Inhibition of PARG indirectly caused PARP hyper-PARylation, interrupting the PARP1 DNA damage repair procedure and resulting in synthetic lethality with HR deficiency (91). It has been determined earlier that the cytotoxicity of PARP-induced DNA trapping is much stronger than the PARPi-induced catalytic activity (92). The inducible-complementarity with a PARG-specific tyrosine clasp and arginine switch explains the competitive inhibition mechanism. In this way, PARG inhibitors suppress replication fork progression and induce cancer cell cytotoxicity, further sensitizing tumor cells to PARP inhibitors. Interestingly, the new PARG inhibitor PDD00017273 exhibits cytostatic rather than cytotoxic effects by causing a replication catastrophe in the interphase instead of mitosis (93). However, the conflicting data on PARG-related rationale for synthetic lethal interaction could not warrant more extensive studies in the future (94, 95). There is still no proposed clinical trial investigating PARG inhibitor and PARPis due to low metabolic stability. However, the newly identified PARG inhibitors COH34 and JA2131 exhibit favorable pharmacokinetic properties and may enter clinical studies (96, 97).

## HR Reactivation

Single-nucleotide frameshift mutations by a short insertion or deletion are the common type of HR-disrupting mutations in BRCA1/2 tumors. The frame restoration of the DNA binding regions by secondary mutations is considered reversible and a cause of PARPi resistance. Several studies have shown the microhomology signatures of the frame reversal mutations (98, 99), suggesting that alternative error-prone mechanisms were responsible for DNA DSB repair in the original HR-deficient cells. Multiple studies have demonstrated the polyclonality of various reversal mutations, thus exerting selection pressure on tumor cells to restore BRCA1/2 activity and reduce PARPi sensitivity.

Studies have identified the methylation and silencing of BRCA1 promoter in sporadic TNBC as a confirmed signature of PARPi sensitivity (100). In addition, duplication and amplification of BRCA2 were identified in PARPi-resistant cell lines, which support the theory that HR deficiency contributes to PARPi resistance (16). A study analyzing PARPi-related patient-derived xenograft models detected the loss of BRCA1 promoter hypermethylation even in germline BRCA1 mutations by mRNA sequence analysis, demonstrating the demethylated or rearranged promoter of BRCA1 in therapy-acquired resistant patient samples (101). Therefore, treatment restoring BRCA1 levels and activity triggers demethylation of the hypermethylated promoter of BRCA1, which is closely associated with PARPi acquired resistance.

Partially reactivated HR, including expression of hypomorphic proteins, is highly associated with RAD51 loading and PARPi resistance (102). Different distribution of segmental mutation varies in BRCA1 and BRCA2 genomic alleles, which directly interacts with clinical outcomes and PARPi sensitivity. BRCT domain affects protein interactions and biological activities. BRCA1 BRCT domain mutation promotes *Alu* elements breakage and truncates transcription by inducing proteasomal degradation. Since BRCTless BRCA1 isoforms are hypomorphs, abundantly expression of isoforms leads to the loss of both BRCA1 and RAD51 foci and clinically manifests as hereditary breast and ovarian cancers (103). BRCA1 BRCT domain-deficient isoforms induce PARPi resistance by promoting HR and avoiding proteasomal degradation. Similarly, exon 11 frameshift mutation-mediated mRNA decay promotes partial PARPi and cisplatin resistance compared with full-length BRCA1. BRCA1 splice produces truncated but hypomorphic proteins through the missing exon 11, which partially compensates for the full-length BRCA1 responding to HR targeting therapies. The interaction of BRCA1-Δ11q-PALB2 is critical in mediating RAD51 *γ*-irradiation-induced foci-(IRIF)and resistance (104). Thus, inhibitors of spliceosome reduce BRCA1-Δ11q levels and resensitize tumors to PARPi treatment. RING domain-deficient BRCA1(Rdd-BRCA1) proteins, retaining hypomorphic activity, facilitate RAD51 foci and promote partial PARPi resistance (105). The C61G mutation in the BRCA1 RING domain is the most frequently initiated missense variant, which is highly associated with ovarian and breast cancer. This C61G mutation triggers resistance to PARPi treatment due to partially reactivated HR (106). Rinske and his group reported that the RING domain-less (RING-less) BRCA1 protein presents partial function of HR by forming a small N-terminal protein of 38aa in genetically engineered mouse models. RING-less BRCA1, carrying the BRCA1185delAG mutation, results from the internal translation of downstream codons and partially restored HR (107). Various allele mutations of BRCA1 proteins form hypomorphic proteins and lead to PARPi resistance.

Early landmark studies demonstrated concomitant mutation of 53BP1 in NHEJ as the cause of HR deficiency and PARPi resistance in BRCA-mutated patients (102). Once 53BP1 is resected or mutated the DNA damage repair exhibits a BRCA1-independent pathway and causes PARPi resistance. Furthermore, several researchers using CRISPR/Cas9 screening of PARPi-resistant cohorts demonstrated a genetic complex comprising REV7, SHLD1, SHLD2, and SHLD3 as active blocker of resection in the HRR pathway (108). The complex is recruited in a 53BP1- and RIF1-dependent manner to DSBs and further blocks nucleases. By partial restoration of HR, tumor cells gain the ability to overcome DSB blockage and acquire resistance to PARPi (109).

## Restoration of Replication Fork Stability

Replication fork is a primary step for checkpoint activation of the cell cycle for DNA damage repair and replication to proceed throughout the S phase. After PARP1 trapping in the DNA binding region, the replication fork is started with two main kinases, ataxia telangiectasia mutated (ATM) and Rad3-related (ATR), by inhibiting the firing of the replicating forks. The kinase ATR, as the primary responder to a stalled replication fork, phosphorylates the downstream signaling cascade, including, reportedly, BRCA1, Mec1, and 53BP1 (110). Stalling the replication fork nucleases of MRE11 and MUS81 leads to fork collapse and chromosomal disturbance, which facilitates genomic instability and arrests the cell cycle of DNA damage repair in BRCA1/2-deficient tumors. RADX is reported as a blocker of the replication fork by inhibiting MUS81 activity and antagonizing the RAD51 remodeling of the fork (111). In addition, several genes, including SMARCAL1, ZRANB3, and HLTF, activate the MRE11-dependent pathway by degrading the fork and remodeling the chromosomes in BRCA1/2-deficient tumors. Loss of RIF1, REV, PTIP, CHD4, JMJD1C is indicated as the cause of PARPi resistance by inhibition of ATR (112). The loss of all the above genes leads to replication fork stalling-related PARPi resistance.

Schlafen 11 (SLFN11) is an indirect factor in replication stress by selectively activating ATPase of the chromatin opening domain to arrest replication fork progression. It selectively binds chromatin through RPA1 and MCM3, instead of phosphorylating ATR or inhibiting the initiation of DNA replication forks under replication stress (113). On the other hand, tumor cells are sensitized by SLFN11 to multiple DNA-targeting drugs, including platinum derivatives, PARPi, inhibitors of topoisomerases, and DNA synthesis inhibitors. By blocking replication forks and inducing replication stress, SLFN11 maximizes cell death during DSB repair (114). Deficiency of SLFN11 due to mutation or epigenetic silencing by inhibitors of HDAC and EZH2 histone methyltransferase leads to tumor progression and resistance to DNA-targeting drugs (115). Consequently, loss of SLFN11 arrests blockage of the replication fork under replication stress in BRCA1/2-deficient tumors and results in PARPi resistance.

## PARPi Combination Therapy

A wide range of mechanisms of acquired resistance to PARPi has been identified in studies. To sensitize tumor cells to PARPi, several agents have been suggested for combination with PARPi.

## PARPi Plus Chemotherapy

Naturally, the DNA repair machinery is conserved and regulated by a series of DNA damage-involved genes when encountered with acute exogenous lesions. Inhibiting DNA repair was brought up as a rationale for the combination of PARPi with chemotherapy. Several chemotherapeutic agents such as cisplatin, carboplatin, paclitaxel, and gemcitabine had been proposed for a phase I clinical trial with PARPi (116). However, the severe side effects of myelosuppression which may be induced by PARP trapping ended the clinical trial. The BROCADE3 trial was designed as a randomized clinical trial including patients with advanced HER2-negative breast cancer with germline BRCA1/2 mutation. The aim was to compare veliparib plus carboplatin and paclitaxel with placebo plus carboplatin and paclitaxel in double blinded and well-balanced groups. In 337 patients treated with veliparib plus paclitaxel, the PFS was 14.5 months [95% confidence interval (CI) =12.5–17.7] *vs* 12.6 months (95% CI = 10.6–14.4) in 172 patients given placebo/chemotherapy (hazard ratio = 0.71; 95% CI = 0.57–0.88; p = 0.02) (3). The median OS was 33.5 months (95% CI = 27.6–37.9) with veliparib/chemotherapy compared to 28.2 months (95% CI = 24.7–35.2) with placebo/chemotherapy (hazard ratio = 0.95; 95% CI = 0.73–1.2, P = 0.67). The results were promising, with a significant improvement in PFS with a combination of veliparib and carboplatin/paclitaxel compared to single chemotherapy. Therefore, the combination of veliparib and carboplatin/paclitaxel exhibits promising anti-carcinogenic effects and can overcome the resistance to veliparib as a PARPi monotherapy in advanced HER2-negative breast cancer.

## PARPi Plus Radiotherapy

Emerging evidence supports a cortical role of PARPis in resistance to radiotherapy by activating DNA repair pathways including HRR and NHEJ. Mutations inducing PARPi resistance, such as inactivation of HR or NHEJ, were validated in sensitizing tumors to radiotherapy (117). DNA-PK and POLɵ have a critical role in DNA-damaging NHEJ and MHEJ pathways, respectively. DNA-PK inhibitors, as DNA-damage inducers, are undergoing clinical trials and have good prospects for prognosis in combination with radiotherapy. Studies have validated the finding that loss of 53BP1/RIF1/REV7/Shieldin/CST pathway in BRCA1-proficient cells restored HR activity, which increased acquired vulnerable radiotherapy sensitivity both *in vivo* and *in vitro* (118). In addition, PARPi has been validated to increase the sensitivity to radiotherapy in BRCAness phenotype. Co-treatment of PARPi and fractioned irradiation exhibited higher efficiency in 2D and 3D cell culture due to a cumulative induction of DNA double-strand breaks (119).

## PARPi Plus Immune Checkpoint Inhibitors

A promising immunotherapy in breast cancer is the newly developed antibodies to programmed cell death ligand-1 (PD-L1) and cytotoxic T-lymphocytic associated protein 4 (CTLA-4) that interferes with robust immune surveillance and escape by exhausting T cells in the tumor microenvironment. Interferon I was reported to be activated by replication stress in the cGAS-STING pathway by clinical PARPi use (120). The activation of interferon I triggers antitumor immunity as well as upregulation of PD-L1 in tumor cells (121). In addition, PD-L1 level in tumors is elevated due to immune stimulation with PARPi treatment (122). As immune checkpoint inhibitors rely on the phosphorylation of the cGAS-STING pathway by using PARPi, it is a persuasive rationale to combine PARPi and PD-L1 antibodies for antitumor therapy. The synergistic effects of combination therapy comprising PARP inhibitors and antibodies against PD-L1 have been identified in breast cancer cell lines and PDXs (123). The confirmed results from Phase II clinical trials reveal that antibodies against PD-L1 (durvalumab or pembrolizumab) in combination with PARPis (olaparib or niraparib) exhibit a good response against germline BRCA1/2-mutated breast cancer and other cancers (124). The hypothesis of an inhibiting combination of ABT-888 with CTLA-4 in BRCA1-deficient tumors is demonstrated by tumor regression and prolonged overall survival (125). Numerous clinical trials investigating CTLA-4 antibodies and PD-L1 antibodies in combination with PARPi are still ongoing with eagerly awaited results (126, 127).

## PARPi Plus Targeted Therapy

HRR pathway deficiency is a critical process in PARPi treatment settings. Some genetic mutations involved in disrupting HRR pathway lead to PARPi sensitivity. The restoration of the replication stalk in PARPi resistance supports the hypothesis that combination of cell cycle checkpoint inhibitors with PARPi works as a promising and overcoming therapeutic strategy (112, 128, 129). ATR and CHK1, as the intra-S-phase checkpoint kinases, are responsible for firing the replication origin by exhausting nuclear RPA and dropping dNTPs to process to scheduled DNA damage repair. Once the checkpoint or replication firing is inhibited DNA damage repair will be interrupted and result in PARPi resistance (130). In several studies, the combination of PARP inhibitors and ATR or CHK1 inhibitors has been identified to block at the intra-S-phase for the reason of replication stalk of chromosomal fragments, resulting in cell death (131). Of note, inhibition of ATM, another DNA damage kinase, leads to progressive DNA damage and accumulated PARylation by activating G2 DNA damage kinase cascade such as ATR, CHK1/2 and WEE1. PARP inhibitors synergistically interact with ATM inhibitors by blocking firing of the replication fork and sensitizing tumor cells to PARP1 trapping, which leads to DNA damage and tumor cell death.

WEE1 is highly expressed and in connection with prognosis in multiple cancers, including breast cancer, ovarian cancer and glioblastoma. As mentioned above, WEE1 functions as a kinase in regulating cell cycle kinases cyclin-dependent kinase1 (CDK1) and CDK2 in S phase and G2/M phase. WEE1 decreases CDK1 expression, subsequently followed by activating replication firing and DSB repair (39). HR is scheduled but weakened by WEE1 inhibitor through phosphorylation of CDK1 in BRCA1/2-deficient tumor cells (132, 133). The combination of PARP and WEE1 inhibitors arrests G2 phase and results in chromosomal aberration and replication stress, which is proved to have antitumor activity in numerous preclinical models (56, 134–136). However, the overlapping toxicity of this combination is a big obstacle demonstrated in clinical trials. The sequential administration in the combination therapy is suggested and has been proven with preserved efficacy and improved tolerance in ovarian cancer cells and PDX models.

CDK inhibitors are a group of inhibitors regulating the activation of cell cycle function by interrupting phosphorylation and transcription. Dinaciclib, as an inhibitor of CDK1, 2, 5, 9, and 12, inhibits the activity of restored HR. By reversing the PARPi resistance, it is synergistically combined with PARP inhibitor, resulting in abrogating MYC expression by suppressing HR restoration, which is confirmed in the PDX model of TNBC (137). CDK4/6 inhibitors have greatly expanded the treatment for ER-positive breast cancer. Multiple studies demonstrated that BRCA genes regulated DNA damage repair and ER expression. In the BRCA-deficient tumors ERα epigenetic silence is abrogated significantly. The anti-estrogen treatment restores the ERα proliferation by reversing G1 arrest, which is a core regulator to genomic instability and apoptosis in HR-deficient cells (138). CDK4/6 inhibitors block the G1 arrest, restore the fork stalking in DNA damage repair, and sensitize the tumors to PARPi treatment. Either in preclinical models or in clinical reports, the combination of CDK4/6 inhibitors and PARP inhibitors in BRCA1/2-mutated, ER-positive patients shows promising results (139).

## Conclusion

Since PARPi were approved by the FDA and EMA as a therapeutic strategy for advanced BRCA1/2-mutant breast cancer, the resistance to PARPi has been the principal obstacle limiting the clinical use in TNBC (140). Genomic instability and aberrant transcription induced by DNA damage repair deficiency are the core mechanisms that form a basis for the treatment of BRCA1/2-deficient or BRCAness-harboring patients. The synthetic lethality mediated by PARP inhibitors in HR-deficient tumors leads to DNA repair deficiency and cell death, due to double loss of chromosomal stability. The mechanisms of PARPi resistance, including increased expression of MDR1, dissociation of PARP1 and PARG, HR restoration, and restoration of replication fork stalling, all reverse the DNA replication pressure and hinder the high sensitivity to PARPi treatment. The promising strategies for TNBC include combining PARPi with chemotherapy, immune checkpoint inhibitors, and cell cycle inhibitors, as well as mutated agents that take a priority in overcoming PARPi resistance (141). Mounting evidence supports combination therapy, which suggests a rational and novel perspective for clinical treatment of TNBC.

## Author Contributions

YH is responsible for organizing and writing. XY and SL collected papers for the topic. YT checked the manuscript and references and gave some remarkable revision suggestions. CL is responsible for the project and final checking. All authors contributed to the article and approved the submitted version.

## Conflict of Interest

The authors declare that the research was conducted in the absence of any commercial or financial relationships that could be construed as a potential conflict of interest.
